# 2-Phenoxyethanol derivatization in ink dating determination

**DOI:** 10.1080/07853890.2021.1897424

**Published:** 2021-09-28

**Authors:** Teresa Argente Leal, Carla Ferreira, Alexandre Quintas, Alexandra Bernardo

**Affiliations:** a Laboratório de Ciências Forenses e Psicológicas Egas Moniz, Centro de Investigação Interdisciplinar Egas Moniz (CiiEM), Egas Moniz Cooperativa de Ensino Superior, Caparica, Portugal

## Abstract

**Introduction:**

2-Phenoxyethanol (PE) is a volatile compound present in the composition of inks. After the deposition in a document, it starts to evaporate over time. This ageing process potentially allow to estimate the date of an ink in a document [1], however this is a complex system where the PE derivatization with different derivatization agents and methods can contribute to improve ink dating validation [2]. The aim of this work is to determine if PE derivatization with MSTFA:TMCS, will increase the sensitivity of the method contributing to the estimation of the ink age for a longer period of time.

**Materials and methods:**

In order to compare peak resolution between PE and derivatized PE, a solution of 50 µg/ml of PE was prepared in hexane. The derivatized sample was prepared using chemical derivatization with MSTFA:TMCS (95:5) at a 80ºC during 30 min. The samples were analysed using GC/MS with an Agilent Technologies 5973 − 6890 N. GC MEGA-5 MS; 0.25µm, 0.32mm, 30 m capillary column was used. Chromatographic analysis was carried out under the following conditions: injection volume 2 µl, split ratio of 2:1 and injection temperature at 280 ºC. The oven temperature program starting at 90 ºC during 8 min, then ramped at 10 ºC min^−1^ to 100 ºC, and increased to 240 ºC at a rate of 30 ºC min^−1^ during 4.67 min. The MS analysis was carried out in SIM mode, which *m/z* of PE was 77, 94, 138 and PE-TMS was 151, 195, 210.

**Results:**

The chromatogram results (Figure 1) showed that PE-TMS has a longer retention time (23.2 min) and a better resolution than PE (retention time of 13.2 min). **Discussion and conclusions:** This preliminary study showed an increase of the sensitivity of the PE-TMS. Our results revealed that PE derivatization with MSTFA:TMCS, might be useful in ink dating determination and can contribute to the decreasing of LOQ. However, more work has to be done. In conclusion, derivatization proves to be promising in the field of ink ageing, allowing getting sensitivity to estimate the age of an ink, in a long period of time.

**Figure 1.**
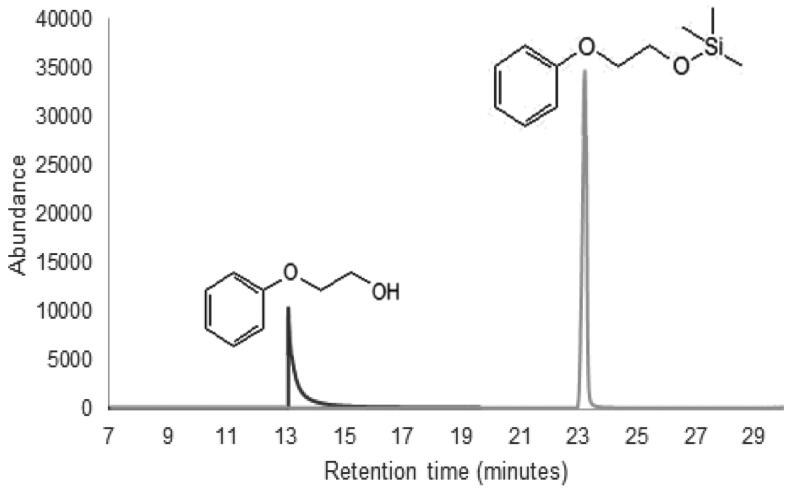
GC-MS chromatogram of PE (tr = 13.2) and PE-TMS (tr = 23.2) carried out in SIM mode using a GC MEGA-5 MS; 0.25µm, 0.32mm, 30 m capillary column.
